# Analysis of ARHGAP4 Expression With Colorectal Cancer Clinical Characteristics and Prognosis

**DOI:** 10.3389/fonc.2022.899837

**Published:** 2022-06-27

**Authors:** Ming-sheng Fu, Shu-xian Pan, Xun-quan Cai, Yuan-xin Hu, Wei-jie Zhang, Qin-cong Pan

**Affiliations:** ^1^ Department of Gastroenterology, Shanghai Fifth People’s Hospital, Fudan University, Shanghai, China; ^2^ Department of Nephrology of Shanghai Fifth People’s Hospital, Fudan University, Shanghai, China

**Keywords:** CRC, ARHGAP4, prognostic, immune, WGCNA

## Abstract

**Background:**

This study aims to analyze the correlation between ARHGAP4 in the expression and clinical characteristics of colorectal cancer (CRC), and the influence of ARHGAP4 expression on the prognosis of CRC, and to evaluate whether ARHGAP4 is a potential prognostic oncotarget for CRC.

**Methods:**

ARHGAP4 was identified using the Gene Expression Omnibus database through weighted gene coexpression network analysis. Using the Gene Expression Profiling Interactive Analysis to perform and analyze the expression and prognosis of ARHGAP4 in CRC. The expression of AGRGAP4 and immune cells was analyzed by the Tumor IMmune Estimation Resource online database. Finally, immunohistochemistry was used to analyze the expression difference and prognosis of ARHGAP4 in CRC and adjacent normal tissues, as well as the relationship between AGRGAP4 expression and clinical features of CRC.

**Results:**

We identified ARHGAP4 that is related to the recurrence of CRC from GSE97781 data. ARHGAP4 has not been reported in CRC. The high expression of ARHGAP4 in select colon adenocarcinoma indicates a poor prognosis by database analysis. In our clinical data results, ARHGAP4 is highly expressed in CRC and lowly expressed in normal tissues adjacent to cancer. Compared with the low-expression group, the high-expression group has a significantly poorer prognosis. In colon cancer, the B-cell, macrophage, neutrophil, and dendritic-cell levels are downregulated after ARHGAP4 gene knockout; the levels of CD8^+^ and CD4^+^ T cells, neutrophils, and dendritic cells are upregulated after the amplification of the ARHGAP4 gene. In addition, ARHGAP4 expression is related to N,M staging and clinical staging.

**Conclusion:**

ARHGAP4 is highly expressed in CRC, and the high expression of ARHGAP4 has a poor prognosis. The expression of ARHGAP4 in CRC is related to the immune cells such as B cells, CD8^+^ and CD4^+^ T cells, macrophages, neutrophils, and dendritic cells. ARHGAP4 is correlated with N,M staging and clinical staging in CRC. ARHGAP4 may be a potential biomarker for the prognosis of CRC.

## Introduction

Colorectal cancer (CRC) is the third most common cancer worldwide. The incidence of CRC in China is rising continuously in recent years; however, most of the patients were still diagnosed in the advanced stage, leading to an unsatisfactory prognosis for them (1).The prognoses of CRC are mainly influenced by the completeness of surgical resection and the pathological stage (2–4). Therefore, there is an urgent requirement to identify potential prognostic biomarkers for the survival improvement of CRC patients.

ARHGAP4 is a member of the Rho GTPase‐activating protein (GAP) family, which recognizes and induces the hydrolysis of guanosine triphosphate (GTP) to produce guanosine diphosphate (GDP). ARHGAP4 is a novel Rho GAP inhibiting axon outgrowth and cell motility (5). ARHGAP4 contains three main functional domains, including Fes/Cip4 homology Bin/amphiphysin/Rvs (F-BAR), Ras homology GTPase activating protein (Rho-GAP), and Src Homology 3 (SH3) domains, among which the Rho-GAP domain is responsible for Rho-GAP activation (6), and the F-BAR domain mediates membrane-related membrane invagination. During the process, it participates in intracellular vesicle transport and endocytosis (7), and the SH3 domain at the C-terminus can bind to proteins containing proline-rich domains and mediate protein–protein interactions (8). The protein encoded by ARHGAP4 can regulate the binding between GTPase and rat sarcoma (RAS) family members. This negative regulation is the small G protein of the Rho family (9), which is related to the occurrence of tumors such as pancreatic cancer (10), liver cancer (11), and lung and prostate cancers (12, 13). Previous research reported that silencing ARHGAP4 promoted the ubiquitination of HDAC2 in the Wnt/β-catenin signaling pathway, thereby inhibiting the activation of β-catenin, increasing the expression of Matrix metalloproteinase2 (MMP2) and Matrix metalloproteinase9 (MMP9), and promoting the invasion and migration of pancreatic cancer cells (14). Na Kang’s study reported that Septin9 is a negative regulator of ARHGAP4; ARHGAP4 promotes tumor migration and epithelial–mesenchymal transition by activating the focal adhesion kinase (FAK)/Src signaling pathway (15). However, so far, there is no report on its function in CRC. In view of this, we plan to study the relationship between ARHGAP4 expression and the clinical characteristics and prognosis in CRC.

Therefore, the purpose of this study is to analyze the correlation between ARHGAP4 expression and the clinical characteristics of CRC and to evaluate the value of ARHGAP4 in the prognosis of CRC, which is a potential biomarker for the prognosis of CRC.

## Materials and Methods

### Data Collection and Clinical Patient

We downloaded the GSE97781 (the patient-derived colonospheres were exposed to six cycles of 5-fluorouracil) series matrix data file from the Gene Expression Omnibus (GEO) public database, a total of 15 sets of transcriptome data, including the pre-treatment group (n=5), post-treatment group (n=5), and recurrence group (n=5) for weighted gene coexpression network analysis (WGCNA). All samples are included in the coexpression network. The soft threshold β is determined by the function “soft $ power estimate,” and the soft threshold is set to 10. Then, the gene module was detected based on the topological overlap matrix (TOM) matrix.

A retrospective analysis was conducted in patients with histologically confirmed colorectal adenocarcinoma who underwent surgical resection in the Department of Gastrointestinal Surgery at Shanghai Fifth People’s Hospital Fudan University between January 1, 2015 and December 31, 2017. The exclusion criteria were as follows: (1) over 90 years old, (2) a clinical confirmation of infectious disease or other diseases that caused systemic inflammation before surgery, (3) patients diagnosed with previous or concurrent malignancies, (4) patients with hematologic disorders, (5) patients with cirrhosis, and (6) patients who received steroid therapy. Lastly, 307 patients were enrolled in this study and informed consent was obtained from all patients or their families. Blood samples were drawn from venous blood within 1 week before the date of surgery by a nurse. The blood samples are tested for the complete blood count and liver function and carcinoembryonic antigen (CEA) value.

The collection of the clinic samples and related experiments were approved by the ethics committee of Shanghai Fifth People’s Hospital Fudan University.

### Construction of Gene Co-Expression Network and Module Detection With Weighted Gene Coexpression Network Analysis

The raw data from the GSE97781 dataset were preprocessed and normalized using the R package “affy” and the “rma” method. Subsequently, the genes were ranked by median absolute deviation from large to small, and the top 5,000 genes were selected for WGCNA using the R package “WGCNA.” The power parameter ranging from 1 to 12 was screened out using the “pick-Soft-Threshold” function. A suitable soft threshold of 8 was selected, as it met the degree of independence of 0.95 with the minimum power value. Subsequently, modules were constructed, and following dynamic branch cutting with a merging threshold of 0.25, 4 modules were obtained. The resulting gene network was visualized as a heat map by selecting all genes based on Topological Overlap Matrix dissimilarity and their cluster dendrogram.

The correlation between module eigengenes and clinical traits were analyzed to identify the modules of interest that were significantly associated with clinical traits. The correlation values were displayed within a heat map. Subsequently, the correlation between the gene significance and the module membership were examined to verify certain module–trait associations. The brown module was correlated the most significantly with CRC recurrence in the heat map. The connectivity of genes was measured by the absolute value of Pearson’s correlation. Genes with high within-module connectivity were considered as hub genes of the modules (cor.geneModuleMembership |MM|>0.95). Hub genes inside a given module tended to have a strong correlation with a certain clinical trait, which was measured by the absolute value of Pearson’s correlation (cor.geneTraitSignificance |GS|>0.8). The correlation analyses were performed using Pearson’s correlation as described in the “WGCNA” package.

### Gene Function Annotation and Gene Set Variation Analysis

Metascape is an intuitive tool for gene annotation and gene list enrichment analysis. In our study, we found that the brown module has a high correlation with recurrence phenotypes by WGCNA analysis. In order to analyze the biological functions and signal pathways involved in the brown module, we use the online Metascape tool to perform module gene function annotation and visualization analysis. Gene ontology (GO) analysis was performed with ARHGAP4 enrichment correlation genes from the brown module. The parameters we selected were Min Overlap= 3, P Value Cutoff= 0.01, and Min Enrichment=1.5 for Pathway & Process Enrichment analysis. Further, we used the R package “Gene Set Variation Analysis (GSVA)” for pathway analysis. By using the ARHGAP4 enrichment correlation genes and setting the P-value to <0.05 and the t-value to >2 as the cut‐off criteria, we performed GSVA in CRC by using the GSVA package in R. The commonly activated/suppressed pathways were identified.

### Expression and Prognosis of ARHGAP4 in Colorectal Cancer

We used the online Gene Expression Profiling Interactive Analysis (GEPIA) tool to perform an analysis of the expression and prognosis of ARHGAP4 in CRC. On the GEPIA homepage, select Expression on Box Plots, enter ARHGAP4 in the gene box, parameter |Log2FC| Cutoff: select 1, p-value Cutoff: select 0.01. In the Datasets Selection (Cancer name), select colon adenocarcinoma (COAD) and rectal adenocarcinoma (READ). Jitter Size automatically matches 0.4; select Match TCGA normal and GTEx data, and select Plots to generate expression results. Continue with Survival Plots, respectively. In the Overall Survival (OS) and Disease Free Survival (RFS), Group Cutoff, select Median; in the Axis Units, select Months; in the Datasets Selection (Cancer name), select COAD and READ, respectively; and finally, select Plots to generate prognostic results.

### The Relationship Between ARHGAP4 and Immune Cells

Tumor IMmune Estimation Resource (TIMER) is a reliable tool that provides systematic evaluations of the infiltration of different immune cells and their clinical impact. In this study, the relationship between ARHGAP4 and the immune cell content was explored through the TIMER database; the correlation between ARHGAP4 expression and tumor immune cell infiltration was analyzed, and the impact of ARHGAP4 gene mutations on tumor immune cells was compared.

### Immunohistochemistry

Tissues were fixed in 4% paraformaldehyde and dehydrated and embedded in paraffin. Approximately 5-μm-thick slices were incubated in 3% H_2_O_2_ in methanol and 5% normal horse serum to minimize nonspecific staining. Sections were incubated at 4°C overnight with the primary antibody: rabbit anti-ARHGAP4 (1:200; 16697-1-AP; Proteintech, Rosemont, IL, USA). Next, the slices were incubated with secondary biotinylated goat anti-rabbit IgG (1:200; SA00004-2; Proteintech, Rosemont, IL, USA) at room temperature for 20–30 min. Subsequently, the sections were stained with diaminobenzidine (DAB), counterstained with hematoxylin for 3 min, and washed in water for 10 min.

Positive reactions were defined as those showing brown signals in the cell cytoplasm. Fields from each slide were examined and photographed under a light microscopy (×20). The immunoreactive score (IRS) (values, 0–12) for each slice was calculated by multiplying the score for staining intensity in four gradations (0, negative; 1, weak; 2, moderate; 3, strong) with the score for the percentage of positive cells in five gradations (0, <1%; 1, 1%–10%; 2, 11%–30%; 3, 31%–70%; 4, >71%), and each specimen was measured in three different magnification fields. Two pathologists independently observed the staining results under double-blind conditions. For statistical analysis, the scores of 0–6 were considered low expression and the scores of 7–12 were considered high expression.

To minimize interobserver variations, all stained slides images were captured using a binocular Leica research light microscope (Leica™ DM2500) at bright field. Images were captured at ×20 magnification using a charged-coupled device (CCD) color video camera (Leica DFC320) attached to a computer system. The field was selected with a good contrast of DAB chromogen and hematoxylin, which is considered a region of interest. All the images were acquired using Leica application software version 3.5.0 (Leica Microsystems, Wetzlar, Germany), which was installed within the computer. Before capturing the images, the color density and white balance were standardized for all images. All the acquired images were saved as Joint Photographic Experts Group (JPEG) format. Then, quantitative analysis was performed on all the images by ImageJ.

ImageJ is a free software; the recent version of ImageJ 1.8.0 version was downloaded from the internet. Open ImageJ, click Plugins -> Macros -> Record … to enable macro recording, click Process -> Batch -> Macro … to enable batch processing based on macro commands, select all images, click Process to start batch processing; after running, copy the integrated option density (IOD) and the ARHGAP4 protein distribution area (Area) of the images to the Excel sheet, and divide the IOD value by the Area to calculate the average optical density (AOD), that is, AOD=IOD/Area, and then analyze and compare AOD value of images.

### Statistical Analysis

All statistical analyses were performed in the R (version 3.6) and SPSS software for Windows (version 25.0). All statistical tests were bilateral, and p<0.05 was statistically significant.

## Results

### Hub Genes Were Screened in Module–Clinical Trait Relationships by Weighted Gene Coexpression Network Analysis

**Figure 1:**
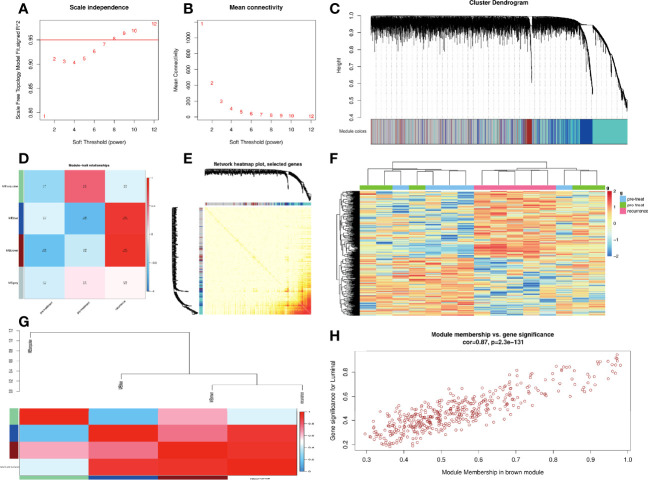
Construction of weighted co-expression network (WGCNA) and WGCNA module analysis. **(A, B)** Soft threshold selection process: scale independence and mean connectivity. **(C)** Cluster module dendrogram. Each color represents one specific co-expression module, and branches above represent genes. **(D)** Heat map of the correlation between clinical traits including pre-treatment, post-treatment, recurrence, and module eigengenes **(E)** Visualizing gene networks. Select genes for the network heat map plot. **(F)** Heat map of the correlation between clinical traits and a module. **(G)** Analysis of the relevancy between blue, brown, gray, and turquoise gene modules and clinical parameters. **(H)** Correlation between the module membership of modules of interest and gene significance with clinical traits in the brown module. Four clinically significant genes with high connectivity were identified in the brown module.

In this study, a total of 4 gene modules were detected from the GSE97781 data by WGCNA analysis, which are blue, brown, gray, and turquoise modules, and their corresponding gene numbers are 680, 423, 2498, and 1399, respectively. We further analyzed the relevancy between gene modules and clinical parameters. The results show that the brown module genes have the most correlation with CRC recurrence. Based the cut-off criteria (|MM|>0.95 and |GS|>0.8), 4 clinically significant genes with high connectivity were identified as hub genes in the brown module, including ARHGAP4, HOXD11, KRT16, and TESC genes (**Figure 1**). Among these four genes, ARHGAP4 has not been reported in CRC so far, which arouses our interest.

### Gene Ontology and Gene Set Variation Analysis

**Figure 2:**
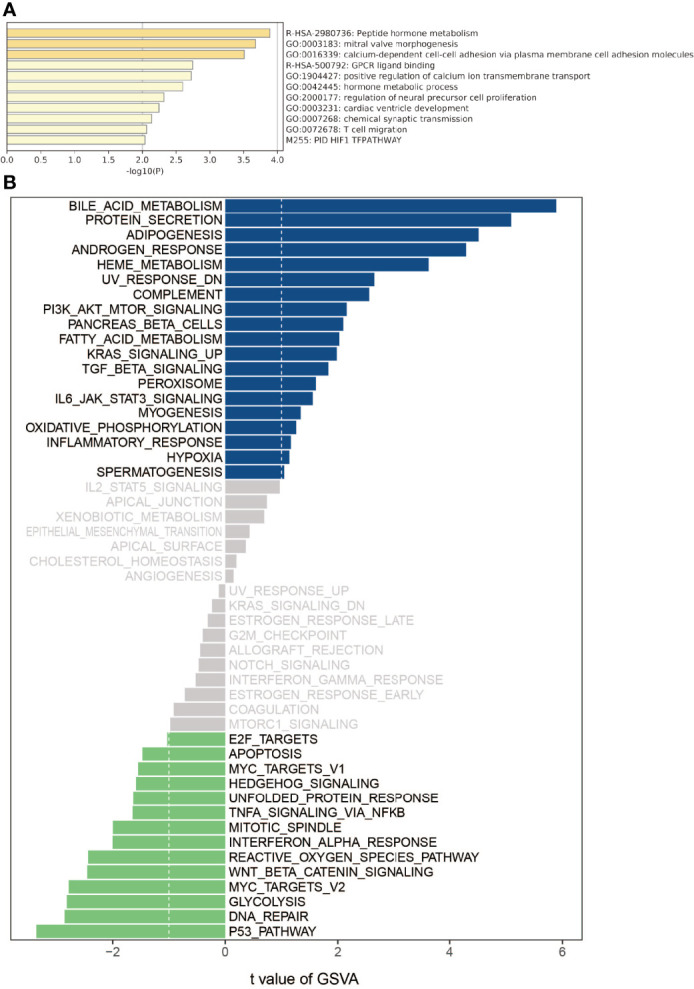
Gene ontology (GO) and gene set variation analysis (GSVA). **(A)** Using the Metascape database for annotation and visualization, GO analysis was performed on the ARHGAP4 enrichment correlation genes from the brown module. **(B)** GSVA divergence bar chart. Using GSVA, the signal pathways of the ARHGAP4 enrichment correlation genes. Bar graph of enriched terms across input gene lists, colored by p-values.

ARHGAP4 enrichment correlation genes were selected from the brown module for GO analysis. The results showed that the ARHGAP4 enrichment correlation genes were mainly related to peptide hormone metabolism, calcium-dependent cell–cell adhesion *via* plasma membrane cell adhesion molecules, and T-cell migration. Further, we conducted the GSVA analysis of ARHGAP4 enrichment correlation genes, and the results showed that the ARHGAP4 enrichment correlation genes were positively correlated with the signaling pathways phosphatidylinositol 3-kinase (PI3K)-protein kinase B (AKT)-mammalian target of rapamycin (mTOR), kirsten rat sarcoma (KRAS), and transforming growth factor-beta (TGF-β) and negatively correlated with Wnt/β-catenin (**Figure 2**).

### Expression and Prognosis of ARHGAP4 in Colorectal Cancer

**Figure 3:**
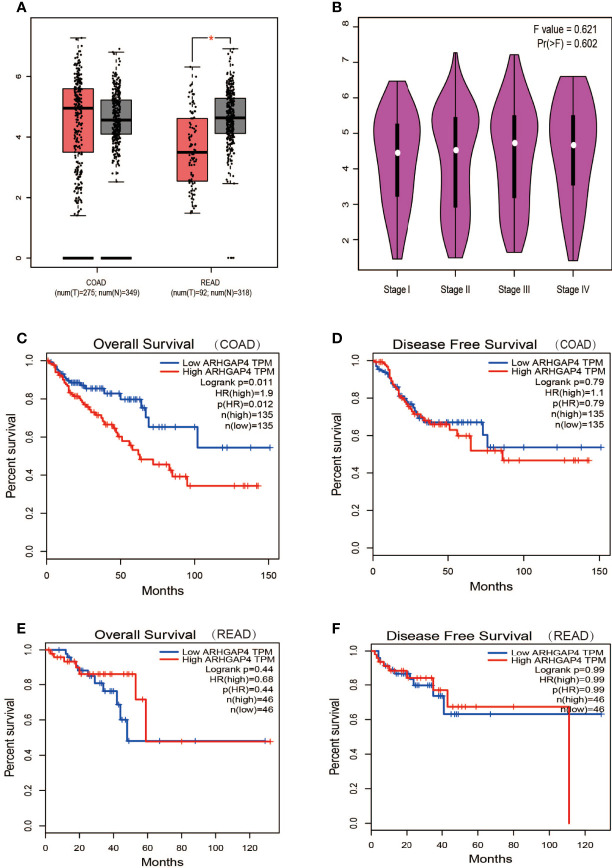
Expression and prognosis of ARHGAP4 in CRC. **(A)** ARHGAP4 is expressed in colorectal adenocarcinoma (COAD) and READ (rectal adenocarcinoma). **(B)** ARHGAP4 is expressed in each clinical stage. **(C, D)** The relationship between ARHGAP4 expression and OS and DFS in COAD. **(E, F)** The relationship between ARHGAP4 expression and overall survival (OS) and disease-free survival (DFS) in READ. *Indicates that the P value is less than 0.05.

Analyzing the expression of ARHGAP4 in COAD and READ through the online GEPIA tool, the results showed that ARHGAP4 was highly expressed in READ compared with normal tissues, and the difference was statistically significant. It is highly expressed in COAD, but there is no significant difference compared with normal tissues (**Figure 3A**). In the clinical stage, the difference in ARHGAP4 expression was not statistically significant (**Figure 3B**). In COAD, the OS of the ARHGAP4 low-expression group was 1.9 times that of the high-expression group (P=0.012), but the DFS of the high-expression and low-expression groups was not statistically significant (**Figures 3C, D**). In READ, compared with the low-expression group, the OS and DFS of the ARHGAP4 high-expression group were not statistically different, as shown in **Figures 3E, F**.

### ARHGAP4 Expression and Immune Cell

**Figure 4:**
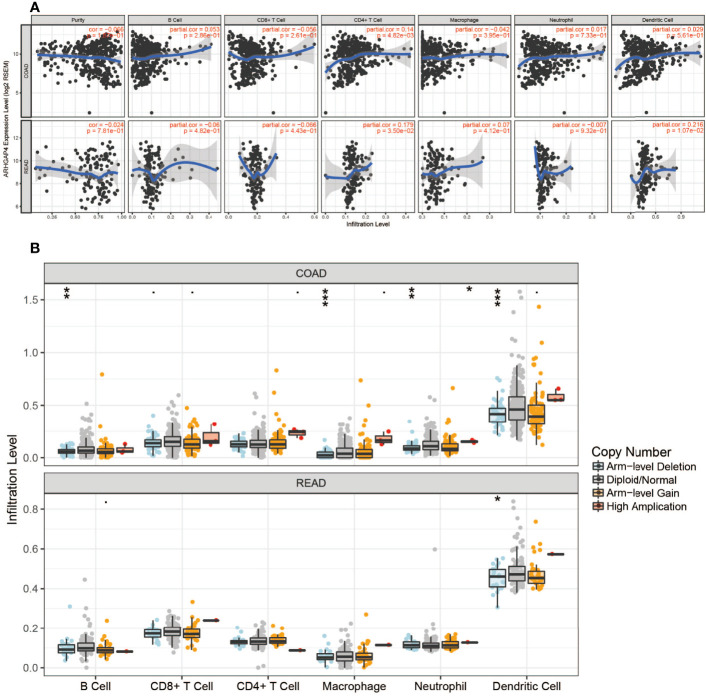
ARHGAP4 gene and immune cells. **(A)** ARHGAP4 expression was highly correlated with CD4^+^ T-cell infiltration in CRC and dendritic cell infiltration in READ. **(B)** After ARHGAP4 gene knockout, the levels of B cells, macrophages, neutrophils, and dendritic cells are downregulated, after the high-amplification ARHGAP4 gene. The levels of CD8^+^ and CD4^+^ T cells, neutrophils, and dendritic cells are upregulated in COAD. *Indicates that the P value is less than 0.05, **Indicates that the P value is less than 0.01, ***Indicates that the P value is less than 0.001.

The expression of ARHGAP4 was highly correlated with the infiltration of CD4^+^ T cells in CRC and with dendritic cells in READ (**Figure 4A**).In colon cancer, after ARHGAP4 gene knockout, the levels of B cells, macrophages, neutrophils, and dendritic cells are downregulated. After the high-amplification ARHGAP4 gene, the levels of CD8^+^ and CD4^+^ T cells, neutrophils, and dendritic cells are upregulated. Meanwhile, it has little effect on immune cells in rectal cancer (**Figure 4B**).

### ARHGAP4 Expression and Prognosis in Pathological Tissues of Patients With Clinical Colorectal Cancer

A total of 307 patients were finally included in the current study, including 183 (59.6%) men and 124 (40.4%) women. The mean age was 70 ± 11 years old (range, 32–90). A total of 186 patients (60.6%) had colon cancer, and the remaining 121 patients (39.4%) had rectal cancer. The evaluation of Tumor-Node-Metastasis (TNM) stages revealed that the clinical pathological diagnoses were 155 patients for stage I–II and 152 patients for stage III–IV. The mean follow-up duration was 33.7 ± 18.8 months (range,0.1–67.7).

**Figure 5:**
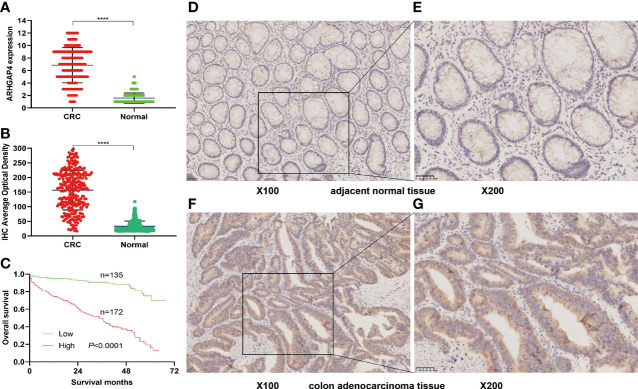
ARHGAP4 expression and prognosis in clinical colorectal cancer (CRC). **(A)** Comparison of ARHGAP4 in CRC tissues and in normal tissues by two pathologists. **(B)** Comparison of ARHGAP4 in CRC tissues and in normal tissues performed quantitative analysis by ImageJ. **(C)** The relationship between ARHGAP4 expression and OS in CRC. **(D, E)** ARHGAP4 is lowly expressed in normal tissues adjacent to CRC. **(F, G)** ARHGAP4 is highly expressed in CRC tissues. ****Indicates that the P value is less than 0.0001.

Immunohistochemistry (IHC) results showed that the IHC score of ARHGAP4 in colorectal adenocarcinoma was significantly higher than the score of adjacent normal tissues by pathologists observed (**Figure 5A**). All the images were performed quantitative analysis by ImageJ, the AOD value of ARHGAP4 in colorectal adenocarcinoma was significantly higher than the AOD value of adjacent normal tissues (**Figure 5B**). ARHGAP4 is negative or lower expression in normal tissues adjacent to cancer and high expressed in colorectal adenocarcinoma tissues (**Figures 5D–G**). In colorectal adenocarcinoma, the ARHGAP4 high-expression group has poor prognosis (**Figure 5C**). **Table 1** shows that ARHGAP4 expression is related to N,M staging and clinical staging. **Table 2** shows that ARHGAP4 expression was negatively correlated with the lymphocyte number and albumin level and positively correlated with the CEA level.

**Table 1:** ARHGAP4 expression and clinical characteristics.

Characteristics		Low	High	X²	*P*-value
		N (%)	N (%)		
Sex	Male	75 (0.41)	108 (0.59)	1.644	0.200
	Female	60 (0.48)	64 (0.52)		
Age	<65	40 (0.47)	45 (0.53)	0.454	0.500
	≥65	95 (0.43)	127 (0.57)		
Site	Rectum	55 (0.46)	66 (0.55)	0.178	0.673
	Colon	80 (0.43)	106 (0.57)		
Size	<5	81 (0.43)	106 (0.57)	0.084	0.772
	≥5	54 (0.45)	66 (0.55)		
T	T0–T2	33 (0.52)	31 (0.48)	1.890	0.169
	T3–T4	102 (0.42)	141 (0.58)		
N	N0	86 (0.49)	88 (0.51)	4.845	0.028
	N1–N3	49 (0.37)	84 (0.63)		
M	M0	122 (0.47)	138 (0.53)	5.995	0.014
	M1	13 (0.28)	34 (0.72)		
Stage	I–II	78 (0.50)	77 (0.50)	5.122	0.024
	III–IV	57 (0.38)	95 (0.63)		

**Table 2:** Correlation between ARHGAP4 expression and blood parameters.

Blood parameters	Low	High	R (Pearson)	*P*-value
White blood cell (*10^9/L)	6.51 ± 2.52	6.64 ± 2.54	0.022	0.703
Neutrophil (*10^9/L)	4.56 ± 2.96	4.65 ± 2.35	0.027	0.634
Monocyte (*10^9/L)	0.44 ± 0.25	0.41 ± 0.20	-0.047	0.409
Lymphocyte (*10^9/L)	1.58 ± 0.68	1.44 ± 0.57	-0.179	0.002
Platelet (*10^9/L)	239 ± 95	242 ± 101	-0.056	0.326
Hemoglobin (g/L)	123 ± 25	119 ± 25	-0.092	0.108
Albumin (g/L)	41.20 ± 5.90	39.50 ± 6.40	-0.141	0.013
Globulin (g/L)	26.60 ± 4.40	26.90 ± 4.20	0.046	0.42
CEA (ng/ml)	15.35 ± 30.47	54.74 ± 177.43	0.139	0.015

CEA, carcinoembryonic antigen.

## Discussion

The incidence of CRC in China is the second among digestive system tumors (16). In 2020, it was estimated that there were more than 550,000 new cases of CRC in China and 283,000 deaths (17).We know that effective treatments for advanced CRC are very limited. Therefore, it is very important to find biomarkers related to the early prognosis and recurrence of CRC.

In this study, we used TOM matrix cluster analysis to screen out gene modules related to CRC recurrence. We detected four gene modules in this analysis, which are blue, brown, gray, and turquoise modules. We further analyzed the relationship between gene modules and traits, it was found that the brown module genes had a high correlation with the recurrence phenotype. Clinically significant ARHGAP4, HOXD11, KRT16, and TESC genes with high connectivity were identified as hub genes in the brown module. Among these four genes, ARHGAP4 is the gene we focused on, which has not been reported in CRC so far.

The GO analysis results showed that ARHGAP4 enrichment correlation genes are mainly concentrated on the peptide hormone metabolism, calcium-dependent cell–cell adhesion *via* plasma membrane cell adhesion molecules, the hormone metabolic process, T-cell migration, and so on. Among them, peptide hormone metabolism and hormone metabolic process pathways are related to tumor progression. They play an important role in maintaining intracellular homeostasis and responding to intracellular and extracellular stimuli. Metabolic changes are one of the important characteristics of tumors. E-cadherin downregulation is associated with certain malignant characteristics, including tumor progression, the loss of differentiation, invasion, and metastasis (18), The study demonstrated that E-cadherin was a metastasis prediction marker and a pre-therapeutic prognostic marker for patients with CRC and hepatic metastases (19).In addition, T-cell migration is related to immunity and is involved in the tumor immune microenvironment to regulate tumor progression and is currently an attractive therapeutic target (20). Previous studies have demonstrated that the migration inhibitory factor (MIF) of macrophages induces cellular proliferation by activating the ERK1-ERK2-MAPK and AKT pathways (21) and suppresses p53-mediated growth arrest and apoptosis (22). Recently, MIF has been proposed as a possible therapeutic target for CRC (23). CRC cells have been reported to secrete MIF at concentrations sufficient to attract T lymphocytes to the tumor (24), and MIF can drive macrophage, neutrophil, and T-cell migration in a chemokine-like manner (25). GSVA results show that ARHGAP4 enrichment correlation genes were positively correlated with PI3K-AKT-MTOR, KRAS, and TGF-β and negatively correlated with Wnt/β-catenin. Previous studies have shown that ARHGAP25 negatively regulates the metastatic potential of CRC cells *via* the Wnt/β-catenin pathway (26).

Database analysis results showed that the high expression of ARHGAP4 in COAD indicates a poor prognosis, which is 1.9 times of the low-expression group. Similarly, our clinical data analysis results show that ARHGAP4 is highly expressed in CRC and lowly expressed in normal tissues adjacent to cancer. Compared with the low-expression group, the high-expression group has a significantly poorer prognosis. In addition, ARHGAP4 expression is related to N,M staging and clinical staging. ARHGAP4 expression was negatively correlated with the lymphocyte number and albumin level and positively correlated with the CEA level. Our previous studies have demonstrated that with a preoperative high neutrophil-to-lymphocyte ratio (NLR), CEA had poorer OS, NLR was an independent predictor of Stage I–II CRC, and the CEA level was an independent predictor of Stage III–IV CRC (27).

Tumor immunity play an important role in gastrointestinal cancer, and immunotherapy strategies are considered to be the most promising direction for the treatment of gastrointestinal tumors (28).In the tumor microenvironment (TME), T-cell failure and cytokine reduction lead to an increased infiltration of regulatory T lymphocytes (Treg) and a high expression of immune checkpoints (ICs) to promote tumor progression (29).CD4^+^ T-cell Tregs negatively regulate the immune response by direct contact to inhibit target cell activation or secrete cytokines TGF-β and IL-10 to inhibit the immune response (30).CD8^+^ T cells are related to tumor metastasis and prognosis (31). The M2 type of macrophages releases matrix metalloproteinases MMP2 and MMP9 to degrade the extracellular matrix, which further stimulates the migration of vascular endothelium and induces angiogenesis and promotes the proliferation and metastasis of tumor cells (32). This study shows that ARHGAP4 was highly correlated with the infiltration of CD4^+^ T cells in CRC. In colon cancer, the levels of B cells, macrophages, neutrophils, and dendritic cells are downregulated after the ARHGAP4 gene knockout. While the levels of CD8^+^ and CD4^+^ T cells and neutrophils are upregulated after high-amplification ARHGAP4.

The limitation of our study is related to the sample size of GSE97781 being too few; in addition, the correlation analysis between the expression of ARHGAP4 in histopathology and blood parameters may have certain limitations. However, our study also has some strengths. Firstly, we found ARHGAP4 as a potential prognostic marker through WGCNA analysis, which has not been reported in CRC. Secondly, we validated the relationship between ARHGAP4 and CRC prognosis to some extent by the database and clinical data analysis. We plan to further study the effect of ARHGAP4 on colon cancer cell proliferation and migration, as well as the molecular mechanism through cell and animal experiments.

## Conclusion

In summary, we found ARHGAP4 from the GSE97781 data by WGCNA analysis, which has not been reported in CRC, so we chose this gene for further study. Database and clinical data results show that ARHGAP4 is highly expressed in CRC; the high expression of ARHGAP4 indicates a poor prognosis. The expression of ARHGAP4 in CRC is related to the immune cells such as B cells, CD8^+^ and CD4^+^ T cells, macrophages, and neutrophil cells. ARHGAP4 is correlated with N,M staging and clinical staging. ARHGAP4 may be a potential new target for the prognosis and treatment of CRC.

## Data Availability Statement

The original contributions presented in the study are included in the article/supplementary material. Further inquiries can be directed to the corresponding authors.

## Ethics Statement

The studies involving human participants were reviewed and approved by ethics committee of Shanghai Fifth People's Hospital Fudan University. Written informed consent for participation was not required for this study in accordance with the national legislation and the institutional requirements.

## Author Contributions

M-sF conceived the study. Y-xH and W-jZ collected the clinical records and follow-up data of all colorectal cancer patients. S-xP processed the clinical data. M-sF conducted the data of GEO, GEPIA, and GSVA and TIMER analysis. X-qC conducted the immunohistochemistry and clinical analysis. M-sF wrote and revised the manuscript for important intellectual content. Q-cP supervised the study. All authors contributed to the article and approved the submitted version.

## Funding

This study was supported by the Shanghai Fifth People’s Hospital Scientific Research Project Fund (No.2020WYZT04) and Natural Science Research Funds of Minhang District, Shanghai (No. 2021MHZ091).

## Conflict of Interest

The authors declare that the research was conducted in the absence of any commercial or financial relationships that could be construed as a potential conflict of interest.

## Publisher’s Note

All claims expressed in this article are solely those of the authors and do not necessarily represent those of their affiliated organizations, or those of the publisher, the editors and the reviewers. Any product that may be evaluated in this article, or claim that may be made by its manufacturer, is not guaranteed or endorsed by the publisher.
